# Specific Heat Capacity of Solar Salt-Based Nanofluids: Molecular Dynamics Simulation and Experiment

**DOI:** 10.3390/ma17020506

**Published:** 2024-01-20

**Authors:** Fahim Mahtab Abir, Donghyun Shin

**Affiliations:** School of Engineering and Technology, Central Michigan University, Mt Pleasant, MI 48859, USA; abir1f@cmich.edu

**Keywords:** thermal energy storage, molten salt, nanofluid, molecular dynamics simulation

## Abstract

In this study, a nanofluid composed of molten solar salt (MSS) and 1.0% SiO_2_ nanoparticles by mass was created and analyzed using differential scanning calorimetry (DSC) to determine its specific heat capacity (SHC). The SHC of the nanofluid was found to be significantly higher than that of pure MSS. The average increase in SHC of the nanofluid with 1.0% SiO_2_ nanoparticles (NPs) loading was found to be 15.65% compared with pure MSS. The formation of nanostructures after doping with NPs may increase the SHC of molten salt (MS) nanofluids, according to certain published research that included experimental confirmation. Nevertheless, no thorough theoretical or computational studies have been conducted to verify the experimental findings related to MSS nanofluid. Molecular dynamics (MD) simulations were conducted in various simulation boxes for different cases to verify the experimental findings and investigate the mechanism behind the enhancement of SHC caused by the addition of SiO_2_ NPs in eutectic MSS. The simulations used pure MSS and mixtures containing NaNO_3_ nanostructures bonded with SiO_2_ NPs. The highest SHC increase of 25.03% was observed when the simulation box contained 13.71% NaNO_3_ nanostructures by weight. The incorporation of NaNO_3_ nanostructures increased the surface area and total surface energy, leading to a positive effect on the SHC of the MSS nanofluid. However, the decrease in the base molten salt’s SHC had a slight negative impact on the overall SHC of the MS nanofluid.

## 1. Introduction

In view of the realities of energy consumption and ecological deterioration, the development and use of new and renewable energy sources are of crucial strategic significance. One of the most promising approaches for converting renewable energy sources into electricity is solar thermal power, which can both generate high-quality electricity and store a large amount of heat without polluting the environment. It is expected that concentrated solar power (CSP) in conjunction with thermal energy storage (TES) will be more cost-effective than CSP alone for dispatchable electricity generation, as TES can effectively achieve a storage capacity of up to 16 h and more. Herrmann et al. [1] and Wu et al. [2] emphasized the fact that one of the most important factors in optimizing the efficiency of generating solar thermal power and lowering the system’s costs is choosing the right heat storage materials. The following advantages make MSS a viable heat transport and storage medium for solar thermal power: thermal stability at high temperatures, a broad range of working temperatures, low viscosity, good thermal stability, low cost, and being environment friendly [3]. Because Carnot’s efficiency improves with the operating temperature, the total efficiency of a power generation system increases with the heat transfer fluid’s operating temperature. As a rise in temperature differential leads to an increase in the buoyancy force that is created, the levels of both convection and conduction, which are kinds of heat transmission, likewise increase.

On the other hand, the SHC of MSS is often rather low. As a result, the thermal storage system required for CSP will be enormous, which will result in a rise in the cost of TES. Hence, it is necessary to increase the SHC of MSS [3]. Adding NPs to MSS in extremely small quantities provides a stable homogenous colloidal suspension of NPs, which is an innovative and low-cost method of increasing the SHC. A nanofluid consists of a base fluid and nanometer-sized particles that range from 1 to 100 nanometers in size that are suspended in the fluid [4]. Almost all MSS-based nanofluids that have been thoroughly researched by many researchers have shown an increase in SHC [3].

Numerous experimental studies on the enhancement of SHC have been published, but it has not been feasible to fully explain the process behind these gains. To explain the observed rise in SHC, several attempts have been made. Three mechanisms for the anomalous augmentation of SHC were first offered by Shin and Banerjee [5]: (i) nanosized particles with greater SHCs, (ii) the energy of the interaction between solids and fluids, and (iii) the formation of a semisolid layer by the stacking of fluid molecules at the surface. However, since most MSS nanofluids only contain a small amount (1% by weight) of NPs, their impact on the effect of SHC in the proposed mechanism is minimal. Moreover, according to the three mechanisms, conventional water-based nanofluids should have shown an increase in SHC; however, most of the studies on water-based nanofluids have reported a decrease in SHC with the addition of NPs. Chieruzzi et al. [6] reported an increase in the SHC of nitrate-based nanofluids, and the agglomeration of NPs was shown to be the reason for the increase in the SHC of MSS nanofluids. However, it could not explain why conventional nanofluids have shown a decrease in SHC even though the agglomeration of NPs was reported in their nanofluids [7]. On the other hand, Mondragón et al. [8] explained the increase in SHC as an exchange of ions between the nitrate ions and NPs, and used Fourier transform infrared spectroscopy (FTIR) to prove their claim. Tiznobaik et al. [9] proposed that salt molecules tend to form nanodendrites at the nanoscale and increase SHC due to the increased specific surface energy linked with nanodendrites’ extraordinarily large surface areas. Later, Rizvi and Shin [10] experimentally visualized the formation of nanodendrites on the surface of a NP by salt molecules via transmission electron micrography. Abir and Shin [11] numerically explored and explained the same observations using MD simulations.

However, very few experimental tools are available to investigate the nanodendrites of MSS-based nanofluids because of their extremely high temperature ranges and their size (i.e., at the nanometer scale). Due to the high melting points of salts, their working temperatures as TES in CSP are around 500 °C. Hence, computational simulation can be an alternative in examining these nanodendrites and their effect on the enhancement of SHC. MD simulation is a powerful technique for computationally elucidating the physical and chemical properties of a wide range of materials at various temperatures, and several studies have already been published on pure MSS mixtures. Hence, in this study, we used the available MD simulation data from the literature, developed nanodendrites in one of the MS boxes, simulated their SHC as a TES, and compared the results with experiments to confirm the reliability of the simulation. Solar salt (NaNO_3_-KNO_3_, 60:40 by weight) was doped with SiO_2_ NPs because this is the most widely used TES candidate in the literature. Most researchers found 1% by weight of SiO_2_ nanoparticles to be appropriate concentration because of the optimal improvement in heat transfer, the stability of the nanofluid, and cost considerations [6,12,13]. Several NaNO_3_ dendrites were embedded in the system while maintaining the salt ratio to resemble the findings in the literature. SHC was computationally calculated by MD simulations and experimentally measured by a DSC. Their results were compared and discussed in order to understand the underlying mechanism behind the enhanced SHC of the MSS nanofluid. Hence, the objective of this investigation was to validate the empirical outcomes by juxtaposing them with the discoveries from the MD simulation and comprehend the underlying mechanism responsible for the augmented SHC of MSS nanofluid.

## 2. Experiment Setup

### 2.1. Nanofluid Synthesis

**Figure 1:**
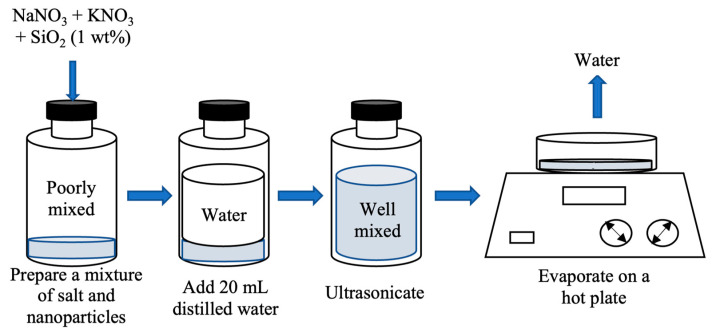
Schematic of the liquid solution method used to produce MSS nanofluids.

All of the samples used in our experiment were synthesized and manufactured according to Shin and Banerjee’s [5] technique. NaNO_3_ and KNO_3_ were procured from LabChem (Zelienople, PA, USA) and Fisher Chemical (Waltham, MA, USA), respectively. Meliorum Technologies (Rochester, NY, USA) was the source of the SiO_2_ NPs that were used in the experiment. NaNO_3_ and KNO_3_ were mixed in a microbalance (SECURA225D, Sartorious, Göttingen, Germany) at a weight ratio of 60:40 to prepare 198.0 mg of the base salt mixture, which included 118.8 mg of NaNO_3_ and 79.2 mg of KNO_3_. In a 25 mL glass vial, 2.0 mg (1 wt%) of SiO_2_ NPs (10–20 nm in size) was combined with the base salt and dispersed in 20 mL of distilled water. The mixture was ultrasonicated for 180 min to ensure proper dispersion of the NPs and dried on a hot plate at 200 °C for 2 h [12]. Figure 1 depicts the procedure of preparing the nanofluid developed by Shin and Banerjee [5] used in this study.

### 2.2. Specific Heat Capacity Determination

A differential scanning calorimeter (DSC 25, manufactured by TA instruments, New Castle, DE, USA) was utilized to determine the SHC of the samples. To verify the repeatability of the samples, fresh samples were generated and analyzed on different days. The experiment was performed in a clean room to eliminate the possibility of the sample being contaminated in any way, and the humidity level in the room was kept below 20% at all times. To prevent any kind of contamination or loss of the samples throughout the DSC testing process, each sample was encased in an aluminum pan with a lid that had been hermetically sealed. To prevent a discrepancy in heat flow from occurring in the DSC, the mass of the sample was changed between 10 and 12 mg. Continuous monitoring of the heat flow within the DSC was performed to ensure that there was neither the presence of moisture nor a chemical reaction.

**Figure 2:**
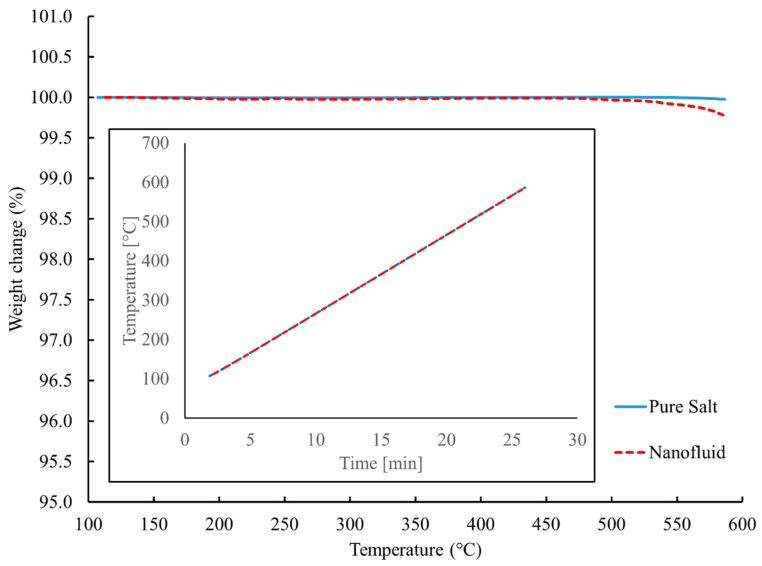
Thermogravimetric analysis (TGA) measurements of the solar salt and solar salt–SiO_2_ nanofluid.

According to the findings of the thermogravimetric analysis (TGA) measurements that are illustrated in Figure 2, the temperature at which the solar salt and the solar salt–SiO_2_ nanofluids decomposed was around 550 °C, which agreed well with the previous literature [12]. As a result, the temperature range for the DSC analysis was selected to be 100–350 °C, at which the thermal stability of the sample could be ensured.

The sample was equilibrated at a temperature of 100 °C, and this temperature was maintained for 5 minutes to ensure that all parts of the sample had the same temperature of 100 °C. After that, this sample was heated until it reached 350 °C at a heating rate of 20 °C/min. Finally, to guarantee the consistency of the signal, the sample was kept at 350 °C for 5 min. In this approach, the SHC of the sample could be calculated using the heat flow of the pan, the heat flow of the pan with sapphire, and the heat flow of the pan containing the sample, in addition to the SHC of sapphire. The equation that was used to calculate the value of the SHC is as follows:

(1) $$c_{p,s}=c_{p,ref}\cdot \frac{\Delta Q_{s}m_{ref}}{\Delta Q_{ref}m_{s}}$$

where the SHC, the difference in heat flow between the sample and the empty pan, and the mass are denoted by the symbols *c_p_*, ∆Q, and *m*, respectively, while the subscripts *s* and *ref* refer to the sample and the sapphire reference material, respectively.

## 3. Setup of the Simulation 

**Figure 3:**
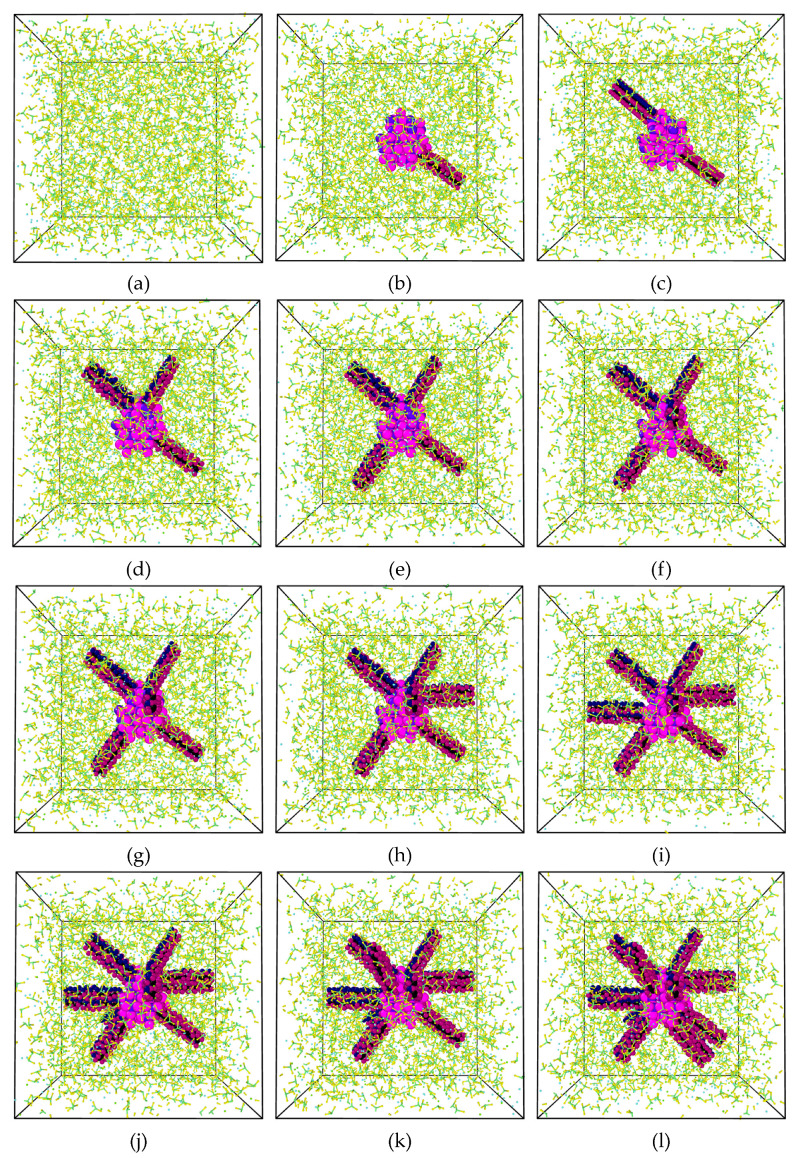

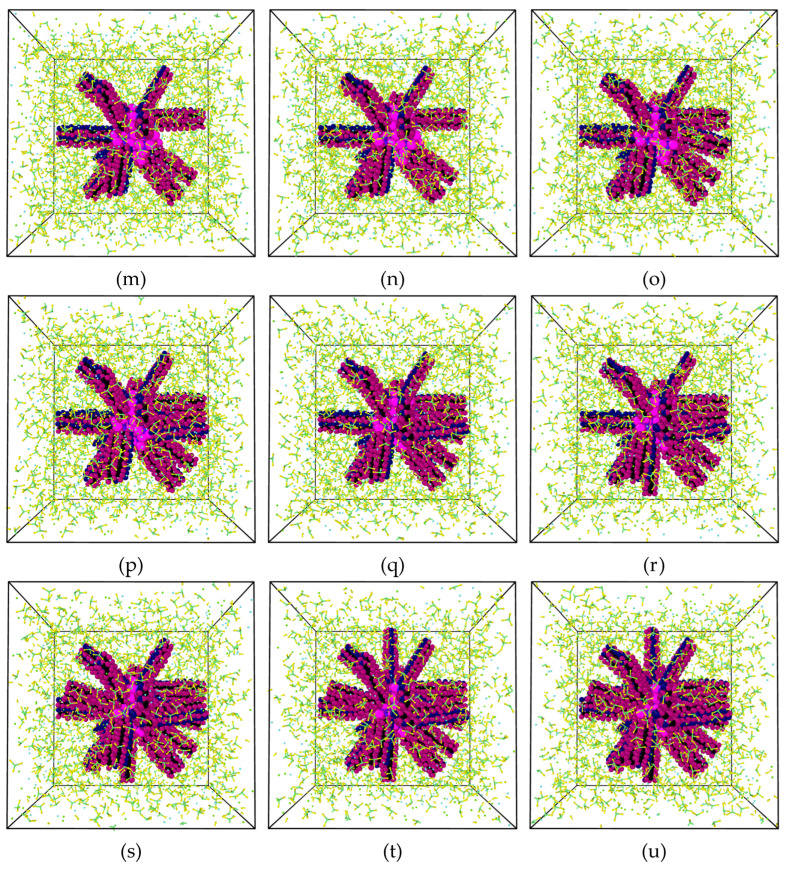
Simulation boxes of (**a**) pure binary nitrate (NaNO_3_–KNO_3_, 60:40 weight ratio) of eutectic solar salt, or solar salt doped with (**b**) 1, (**c**) 2, (**d**) 3, (**e**) 4, (**f**) 5, (**g**) 6, (**h**) 7, (**i**) 8, (**j**) 9, (**k**) 10, (**l**) 11, (**m**) 12, (**n**) 13, (**o**) 14, (**p**) 15, (**q**) 16, (**r**) 17, (**s**) 18, (**t**) 19, and (**u**) 20 NaNO_3_ nanostructures attached to a SiO_2_ NP.

Numerous simulation boxes were generated with the help of *MedeA* (Version 3.1, a protected brand of Materials Design, Inc., San Diego, CA, USA). Each simulation was given a box with the initial dimensions of 4.91 nm × 4.91 nm × 4.91 nm, and allowed us to adjust the volume to maintain a constant pressure inside the box. A simulation box was first built with a pure mixture of NaNO_3_ and KNO_3_ with a weight ratio of 60:40, and this included 7095 atoms in total (909 molecules of NaNO_3_ and 510 molecules of KNO_3_). This is seen in Figure 3a. The simulation boxes of the development of 20 NaNO_3_–KNO_3_ systems are shown in Figure 3b–u, where 1 to 17 NaNO_3_ nanostructures (each nanostructure containing 150 atoms) was bound to a single SiO_2_ NP, which was situated in the middle of the simulation boxes.

In this simulation investigation, the large-scale atomic/molecular massively parallel simulator, which is popularly known as LAMMPS and created by Sandia National Laboratory [14], was used to determine the thermophysical parameters (e.g., SHC and density) of the eutectic MSS (NaNO_3_–KNO_3_) and its nanofluid. The Lennard–Jones (LJ) potential is often used for modeling MSS systems because of its lower computing costs [15] and because it maintains the stability of all systems throughout extended computing steps. According to a prior study [16], the intermolecular atomic mobility between two unbonded atoms in MSS was calculated using the LJ potential. Therefore, because of its simplicity and economical computational cost, LJ potential was used to describe the molten salt system in this study. The density and SHC of the MSS and its nanofluids were calculated using Equation (2)’s LJ potential and long-range Coulombic force.

(2) $$E=\frac{Cq_{i}q_{j}}{r}+4\varepsilon \left[{\left(\frac{\sigma }{r}\right)}^{12}-{\left(\frac{\sigma }{r}\right)}^{6}\right]\;where\;r<r_{c}$$

In Equation (2), *E* denotes the potential energy that might be created between two atoms, *C* represents a constant of the conversion of energy, the charge of two atoms (*i* and *j*) is represented by *q*, the distance between those atoms is denoted by *r*, the cut-off gap between them is denoted by *r_c_*, *ε* denotes the potential wall’s depth, and *σ* refers the fixed gap when the potential between two particles is zero. Long-range Coulombic interactions in the simulation framework were computed with an accuracy level of 10^−4^ using the typical Ewald summation [17]. In each of the systems that was made for the simulation, the computation time step for obtaining a stable result was 0.5 *fs*, and as a minimization strategy, the Polak–Ribiere form of the conjugate gradient was applied [18]. To determine the parameters of the interatomic interaction of the LJ potential between distinct atoms, the Berthelot mixing rule was utilized [19]. For intramolecular atomic motion or bound contact, all the simulation frameworks used harmonic style bond potential force, a harmonic-like angle potential force, and a cvff-like torsional potential force. Every simulation adhered to a system trajectory that was compatible with the microcanonical ensemble (NVE), and the isothermal–isobaric ensemble (NPT) was developed by applying Nose–Hoover temporal integration on the motion equations that were non-Hamiltonian [17]. Prior research [20] was used to establish the initial potential parameters which were modified in order to fit the present system. Table 1 displays the potential parameters that were utilized in each of the simulations conducted for this investigation.

**Figure 4:**
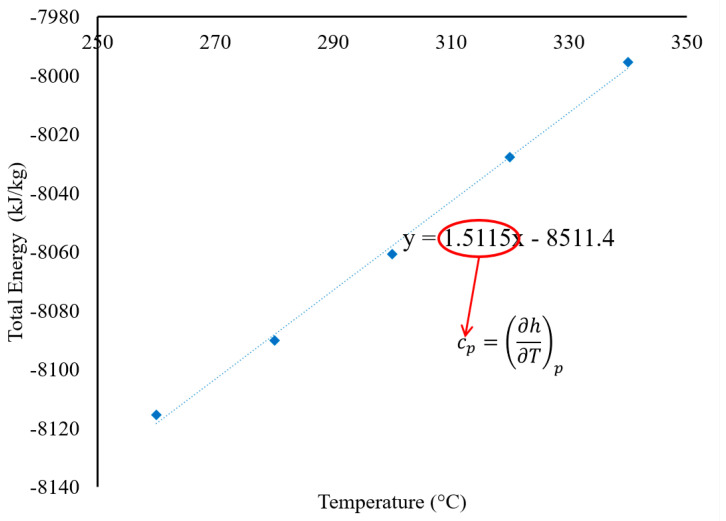
Determination of the SHC of pure solar salt by using the slope (total energy versus temperature).

To compute the SHC of the eutectic MSS and its nanofluid, the overall combined energy (kinetic and potential energy) of the eutectic MSS and its nanofluid was estimated at various temperatures between 260 °C (533 K) and 340 °C (613 K). The slope ($c_{p}={\left(\frac{\partial h}{\partial T}\right)}_{p}$, where *c_p_* refers to SHC, *h* refers to enthalpy, and *T* refers to temperature) from the total energy versus temperature graph was then calculated to obtain the SHC of the pure eutectic salt and its nanofluid, as shown in Figure 4. To reduce the massive computational cost and time, the MD simulation system required massive supercomputing capacity and systems with high-performance supercomputers to determine the quantity of atoms (molecules). All simulations were performed at Michigan State University’s high-performance computing center (HPCC).

The whole process of calculating the thermophysical characteristics (density, SHC, etc.) using MD simulation is as follows. Setting up the intended system in the simulation environment is the first step. Minimizing the energy of the system is the next step. During this stage of the process, the coordinates of atoms will go through an iterative process of modification to reduce the amount of potential energy until a regional minimum value is achieved. Atoms are kept from colliding with or becoming too near to one another in this process, which would otherwise lead to instability in the simulation. The next phase is the NVE phase, which is related to equilibration of the microcanonical ensemble. During the NVE phase, the quantity of atoms, the energy, and the volume of the system stay constant, while the temperature fluctuates. Again, the NVE stage is necessary to establish whether or not the system (such as the simulated box) is steady. When the temperature is steadily raised, the density of the system may be calculated (NVE) under these conditions. The isothermal–isobaric ensemble (NPT) is utilized to guide the system to the minimum temperature running equilibration after confirming its stability. The NPT indicates that the number of atoms, the pressure, and the temperature of a system stay constant as its volume varies. As the system converges, the mass density is calculated by averaging the values of density [21,22]. Thermostatting (velocities of the particles) and barostatting (dimensions of the simulation box) are used to modify the pressure and temperature, respectively. After using the scaling of velocity (temperature) to raise the temperature of the system to an acceptable level, the combined energy, temperature, and density values may be determined. Once the total energy, density, and temperature converge, the computation phase must be finished. If every value fails to converge, the preceding operation has to be repeated (e.g., minimization, equilibration of NVE or NPT). At different temperatures, the overall combined energy (kinetic and potential energy) was computed, and the gradient from the total energy versus temperature graph was utilized to measure the SHC of the pure eutectic salt and its nanofluids [15]. The visualization stage is necessary after the calculation to inspect the location of every atom (molecule) or the creation of every structure to decide if it is steady or not. Each simulation box was represented graphically by OVITO [23].

## 4. Results and Discussion

### 4.1. Determination of the Specific Heat Capacity by Experiments

**Figure 5:**
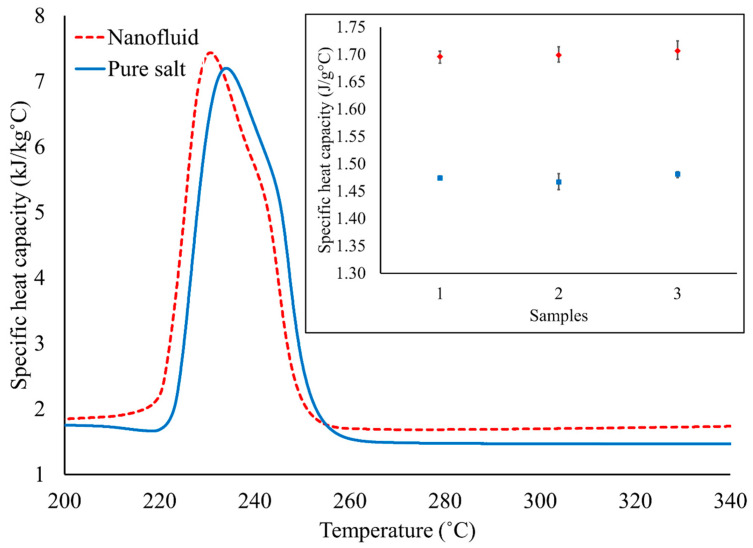
Variations in the SHC of pure solar salt and solar salt doped with 1.0% (weight percentage) of SiO_2_ NPs with temperature.

According to Equation 1, the SHCs of pure solar salt and solar salt doped with 1 wt% SiO_2_ NPs were computed, as shown in Figure 5 and Table 2. As observed, the average SHC of pure solar salt was around 1.47 kJ/(kg·°C) at 300 °C, which is comparable with the values reported in the prior literature (1.48 kJ/(kg·°C) in the liquid phase) [12]. The uncertainty between the measured value and value in the literature is just 0.68%, which indicates that the measured value is reliable. Since solar salt is used in TES systems as a sensitive heat material in the liquid phase, only results between 260 °C and 340 °C have been taken into consideration. All the results were taken at 300 °C, as this can be considered a molten (liquid) state for solar salt. After doping the solar salt with 1 wt% SiO_2_ NPs, the average SHC was found to be 1.70 kJ/(kg·°C), as shown in Table 2. Therefore, the average augmentation in SHC was found to be 15.65% in comparison with the pure solar salt. Table 2 also shows that every sample ran for three cycles so that the SHC value of each sample found from the SDSC was justified to have repeatability and reliability. The average SHC was finally computed by averaging the SHC values of three samples of pure solar salt and three samples of the solar salt’s nanofluid to ensure repeatability and reliability. The standard deviation was calculated to be 0.007 and 0.005 for the SHC of pure solar salt and the solar salt nanofluid, respectively, which was very reasonable. It is clear from the values in Table 2 that the SHC of MSS can be increased by adding a minute concentration of NPs into it. The mechanism behind this increase in SHC is a hot topic of research. Recently, Rizvi and Shin [10] gave a hypothesis that the development of nanostructures in the MSS nanofluid could be the reason behind the enhancement of the SHC of MSS nanofluid. However, it is very difficult to explain this mechanism experimentally. Therefore, MD simulation was used to explain the mechanism behind the increase in SHC.

### 4.2. Analysis of the Mechanism by Molecular Dynamics Simulation

MD simulation was utilized to determine the SHC and density of pure solar salt and the SiO_2_-seeded solar salt with various numbers of NaNO_3_ nanostructures. As shown in Table 3, the density of pure solar salt was found to be 1.87 g/cm^3^ in the MD simulation, and the value in the literature is 1.804 g/cm^3^ [24]. The relative error between the measured value from the MD simulation and the value in the literature was 3.66%, which was less than 5%. Therefore, it can be said that the value of density found in the MD simulation was reliable. Again, according to Table 3, the SHC of pure solar salt was determined to be 1.5115 kJ/(kg.°C) by using MD simulation, and the previous literature [12] reported that the SHC of pure solar salt was found to be 1.48 kJ/(kg.°C) by DSC. In this case, a relative error of 2.13% between the measured value from the MD simulation and the value in the literature was calculated, which was less than 5% and thus the SHC found by the MD simulation was reliable as well.

**Figure 6:**
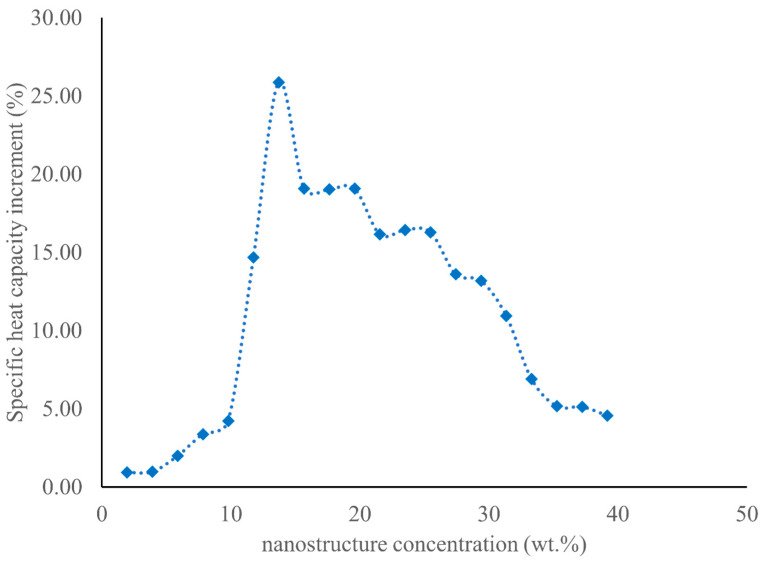
Variation in the increase in SHC (%) with the number of nanostructures (wt%).

MD simulation was used to determine the SHCs of NaNO_3_–KNO_3_ (60:40 by mass) or solar salt in conjunction with different weight percentages of NaNO_3_ nanostructures coupled to a SiO_2_ NPs (1 wt%). As seen in Figure 6 and Table 4, up to 5% weight concentration of the NaNO_3_ nanostructures in the MSS nanofluid system, there was a negligible increase in the SHC of the MSS nanofluid. There was a small increase (~5%) in the SHC of the MSS nanofluid discovered when the weight percentage of the NaNO_3_ nanostructures was between 5% and 10%. The SHC of the MSS nanofluid system was found to be significantly improved when the weight percentage of the nanostructures fell within the range of 10% to 15%. When the weight percentage of the NaNO_3_ nanostructures reached 13.71% compared with the entire weight of the MS nanofluid system, it was discovered that the improvement in SHC was at its greatest possible level of 25.87%. The maximum enhancement of SHC was also reported to be 25.03% in previous literature [12], in which the solar salt was mixed with 1.0 wt% of SiO_2_ NPs. This proves that the maximum 25.87% increase in SHC found from the MD simulation is reasonable. As seen in Table 2, the maximum increase in SHC in the experiment was determined to be 15.65% by using a differential scanning calorimeter (DSC) when the solar salt was doped with 1.0 wt% of SiO_2_ NPs. As seen in Table 4, a 15.65% increase in the SHC was found when the weight percentage of the nanostructures in the MSS nanofluid was either between 11.76% and 13.71%, or between 25.47% and 27.43%. With an increase in the weight concentration (up to 13.71 wt%) of the NaNO_3_ nanostructures, the SHC of the MSS nanofluid increased. Understanding of the surface energy of the nanostructures may shed light on the phenomenon of increased SHC. The nanostructures, similar to NPs, possess an extremely high specific surface area. According to the research [9,10,11] that has previously been published, the effect of the surface energy on SHC can be greatly amplified when there is a large specific surface area. This is due to the fact that a higher surface area increases the ratio of surface atoms to interior atoms for a given volume. The improvement in the SHC of MS nanofluids has been rationalized in many other published works [9] using the same line of thinking. The enhanced SHC of the MSS nanofluid began to drop at a point when there was a further rise in the mass concentration of the NaNO_3_ nanostructures to above 13.71 weight percent. However, this drop is not a particularly dramatic one. The enhanced SHC of the MSS nanofluid gradually decreased with an increasing weight percentage of NaNO_3_ nanostructures. Rizvi and Shin claimed in published research [10] that the electrostatic attraction between the salts in a binary mixture, which results in microsegregation between the salts, is thought to be the source of these nanostructures. Salt that has a higher zeta potential (NaNO_3_ in the present study) is more likely to be drawn to the NPs and has a greater tendency to gather around them. This causes the composition of the mixture that is around the NPs to become unstable. This salt then nucleates in a non-uniform manner on the surface of the NPs due to the process of thermophoresis, which results in the development of dendritic nanostructures. Therefore, with an increase in the creation of NaNO_3_ nanostructures, there is a continuous shift in the eutectic ratio of the MSS. As the weight concentration of the nanostructure of NaNO_3_ rises, the quantity of NaNO_3_ that is present in the eutectic MSS drops. Lee and Jo [25] demonstrated that the SHC of a eutectic NaNO_3_–KNO_3_ salt with a molar ratio of 50:50 was 1.481 J/g·K, but the value of SHC for a eutectic NaNO_3_–KNO_3_ salt with a molar ratio of 34:66 was only 1.285 J/g·K. It is reasonable to expect that the SHC of a eutectic MSS will decrease in proportion to the reduction in the quantity of NaNO_3_ present in the eutectic MSS. Similarly, in the case of a binary carbonate salt, Jo and Banerjee [26] and Araki et al. [27] revealed that a eutectic Li_2_CO_3_–K_2_CO_3_ salt (62:38 molar ratio) was predicted to have the maximum SHC. However, when the mol% of Li_2_CO_3_ in the eutectic Li_2_CO_3_–K_2_CO_3_ salt was lowered, its SHC declined. Therefore, according to the literature [25,26,27] cited above, even though nanostructures tend to increase the SHC of the MSS nanofluid by increasing the specific surface area, the lower SHC of the base MSS hinders the enhancement of the overall SHC of the MSS nanofluid.

## 5. Conclusions

In this study, a nanofluid composed of molten solar salt (MSS) and 1.0% SiO_2_ nanoparticles (NPs) by mass was created and analyzed using differential scanning calorimetry (DSC) to determine its specific heat capacity (SHC). The SHC of the nanofluid was found to be significantly higher than that of pure MSS. To ensure accuracy and reproducibility, multiple samples of the pure MSS and nanofluid were tested. The average increase in SHC of the nanofluid with a loading of 1.0% SiO_2_ NP was found to be 15.65% compared with pure MSS.

Molecular dynamics (MD) simulations were conducted in various boxes to investigate the mechanism behind the enhanced SHC caused by the addition of SiO_2_ NPs in eutectic MSS. The simulations used pure MSS and mixtures containing NaNO_3_ nanostructures bonded with SiO_2_ NPs. The highest increase in SHC of 25.03% was observed when the simulation box contained 13.71% NaNO_3_ nanostructures by weight. The incorporation of NaNO_3_ nanostructures increased the surface area and total surface energy, leading to a positive effect on the SHC of the MSS nanofluid. However, a decrease in the base molten salt’s SHC had a slight negative impact on the overall SHC of the MSS nanofluid.

The increase in the SHC of salt-based nanofluids could significantly reduce the cost of thermal energy storage in concentrated solar power (CSP) system and subsequently lower the cost of electricity generation. A thorough understanding and comprehension of the mechanism accountable for the enhanced SHC can guide and improve cost-cutting efforts in this area.

## Figures and Tables

**Table 1:** Parameters of LJ potential implemented in the simulations.

Atom Symbol	*q*	*ε* (kcal/mol)	*σ* (Å)
N (nanostructure)	+0.50	0.167	3.700557
Na (nanostructure)	+0.00	1.607	2.005129
O (nanostructure)	−0.50	0.228	3.02302
O (nanoparticle)	−0.50	0.150	3.11814
Si (nanoparticle)	+0.00	0.300	3.8270
K (salt)	+1.00	5.451	3.379229
N (salt)	+1.10	0.167	3.700557
Na (salt)	+1.00	1.607	2.005129
O (salt)	−0.70	0.228	3.02302

**Table 2:** SHC of pure solar salt and solar salt doped with 1.0 wt% of SiO_2_ NPs at 300 °C.

Specific Heat Capacity (kJ/kg °C)								
	Solar Salt				Solar Salt + 1.0 wt% SiO_2_			
Sample No.	Cycle 1	Cycle 2	Cycle 3	Average	Cycle 1	Cycle 2	Cycle 3	Average
1	1.478	1.471	1.474	1.474	1.706	1.698	1.684	1.696
2	1.482	1.466	1.453	1.467	1.714	1.697	1.686	1.699
3	1.486	1.475	1.483	1.482	1.725	1.704	1.691	1.707
Average	-	-	-	1.474	-	-	-	1.700
St. deviation	-	-	-	0.007	-	-	-	0.005
Enhancement (%)	-	-	-	-	-	-	-	15.65

**Table 3:** Simulated results for the density and SHC of pure solar salt.

Simulation System	Density (g/cm^3^) at 573 K or 300 °C	Value in the Literature (g/cm^3^)	SHC (kJ/kg °C) at 573 K or 300 °C	Value in the Literature (kJ/kg °C)
Pure solar salt	1.87	1.804 [24]	1.5115	1.48 [12]

**Table 4:** Simulation results for the SHC of pure solar salt, and solar salt doped with different weights (%) of nanostructures connected to the SiO_2_ NPs.

System	NS (wt%)	Specific Heat Capacity (kJ/kg °C)	Enhancement (%)
Pure solar salt		1.5115	
Solar salt with SiO_2_ + 1NS	1.96	1.5255	0.93
Solar salt with SiO_2_ + 2NS	3.92	1.5263	0.98
Solar salt with SiO_2_ + 3NS	5.88	1.5416	1.99
Solar salt with SiO_2_ + 4NS	7.84	1.5626	3.38
Solar salt with SiO_2_ + 5NS	9.80	1.5753	4.22
Solar salt with SiO_2_ + 6NS	11.76	1.7334	14.68
Solar salt with SiO_2_ + 7NS	13.71	1.9026	25.87
Solar salt with SiO_2_ + 8NS	15.67	1.7999	19.08
Solar salt with SiO_2_ + 9NS	17.63	1.7991	19.03
Solar salt with SiO_2_ + 10NS	19.59	1.7998	19.07
Solar salt with SiO_2_ + 11NS	21.55	1.7556	16.15
Solar salt with SiO_2_ + 12NS	23.51	1.7599	16.43
Solar salt with SiO_2_ + 13NS	25.47	1.7577	16.29
Solar salt with SiO_2_ + 14NS	27.43	1.7171	13.60
Solar salt with SiO_2_ + 15NS	29.39	1.7108	13.19
Solar salt with SiO_2_ + 16NS	31.35	1.6769	10.94
Solar salt with SiO_2_ + 17NS	33.31	1.6159	6.91
Solar salt with SiO_2_ + 18NS	35.27	1.5896	5.17
Solar salt with SiO_2_ + 19NS	37.23	1.5889	5.12
Solar salt with SiO_2_ + 20NS	39.19	1.5805	4.57

## Data Availability

Data are available on request.
